# Wellbeing in the Making: Peoples’ Experiences with Wearable Activity Trackers

**DOI:** 10.1186/s13612-016-0042-6

**Published:** 2016-06-14

**Authors:** Evangelos Karapanos, Rúben Gouveia, Marc Hassenzahl, Jodi Forlizzi

**Affiliations:** Cyprus University of Technology, 3036 Limassol, Cyprus; Madeira-ITI, University of Madeira, Funchal, Portugal; Experience and Interaction, Folkwang University of the Arts, Essen, Germany; Carnegie Mellon University, Pittsburgh, PA USA

**Keywords:** Personal informatics, Wearable activity trackers, User experience

## Abstract

**Background:**

Wearable activity trackers have become a viable business opportunity. Nevertheless, research has raised concerns over their potentially detrimental effects on wellbeing. For example, a recent study found that while counting steps with a pedometer increased steps taken throughout the day, at the same time it decreased the enjoyment people derived from walking. This poses a serious threat to the incorporation of healthy routines into everyday life. Most studies aim at proving the effectiveness of activity trackers. In contrast, a wellbeing-oriented perspective calls for a deeper understanding of how trackers create and mediate meaningful experiences in everyday life.

**Methods:**

We present a study of real life experiences with three wearable activity trackers: *Fitbit*, *Jawbone Up* and *Nike* + *Fuelband*. Using need fulfillment as a theoretical lens, we study recent, memorable experiences submitted by 133 users of activity trackers.

**Results:**

We reveal a two-dimensional structure of users’ experience driven by the needs of *physical thriving* or *relatedness.* Our qualitative findings further show a nuanced picture of the adoption of activity trackers and their impact on wellbeing. For instance, while reflection about own exercising practices lost its relevance over time, users continued to wear the tracker to document and collect their runs. More than just supporting behavioral change, we find trackers to provide multiple psychological benefits. For instance, they enhance feelings of *autonomy* as people gain more control about their exercising regime. Others experience relatedness, when family members purchase a tracker for relatives and join them in their efforts towards a better, healthier self.

**Conclusions:**

The study highlights that activity trackers can be more than “tools” to change behavior. Through incorporation in daily life, they offer new social experiences, new ways of boosting our self-esteem and getting closer to our ideal selves.

## Background

The market of wearable activity trackers, such as *Fitbit*, *Jawbone up*, and *Nike* + *Fuelband*, has grown to a volume of $330 million in 2013 (NPD, 2013). This success originates from a trend, which started more than three decades ago: the rise of “wellness, self-help, and holistic health ideals, […] and the popularity of jogging and other fitness activities like aerobics, bicycling, and running” (Berryman 2010, p. 9). In the 1980s, at least for some, exercise became a fashionable part of life style. This was fueled further by the broad promotion of the beneficial effects of exercise on chronic cardiovascular and respiratory diseases as well as diabetes. In 2007, exercise was deemed the “wonder drug” by the presidents of the American Medical Association and the American College of Sports Medicine (Berryman 2010). Since chronic diseases account for nearly 40 % of mortality and 75 % of health care costs, with obesity alone being responsible for an estimated 12 % of the health spending growth in the U.S. (RWJF Health Policy Snapshot 2011), any potential means to motivate people to increase physical exercising in everyday life appears highly desirable. In this sense, activity trackers are not only gadgets to support healthier lifestyles, but tools to increase patient-driven prevention and to reduce cost.

**Fig. 1:**
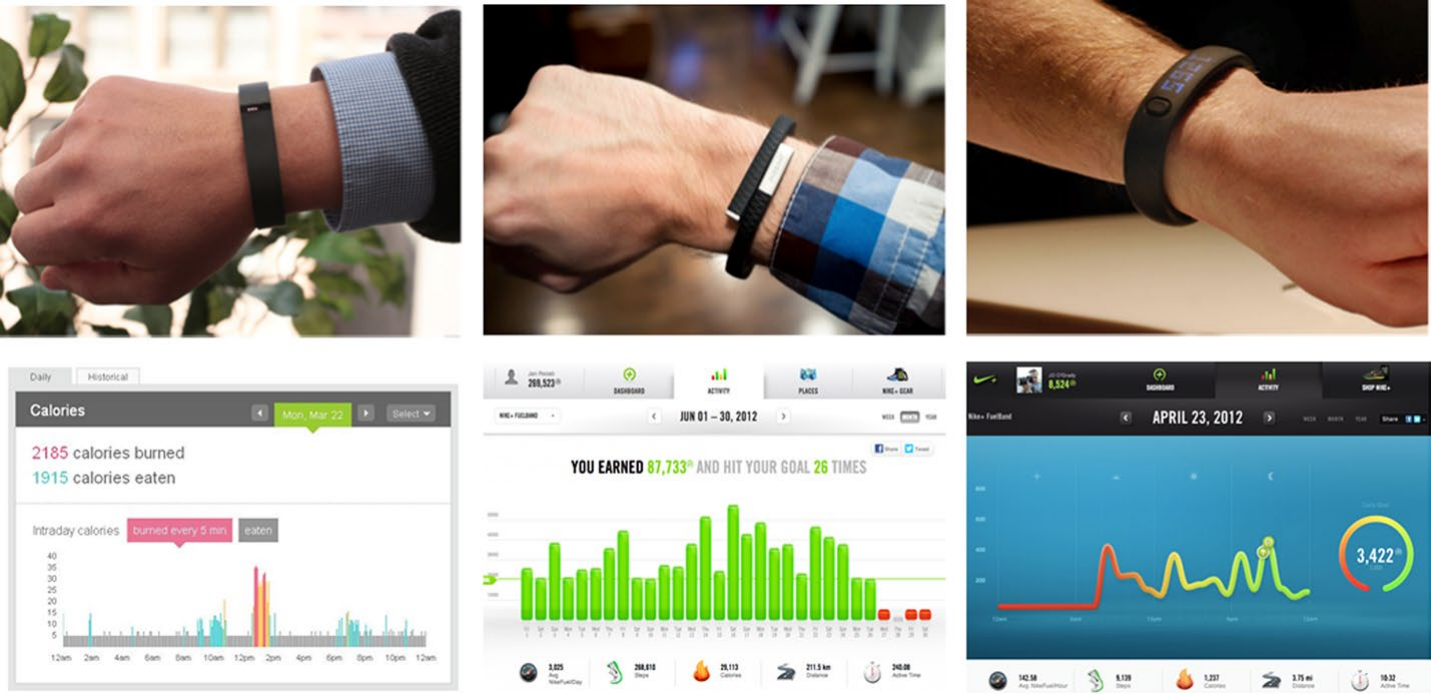
The three activity trackers with feedback visualisations—*Fitbit* (*left*), *Jawbone Up* (*center*), *Nike*+ *Fuelband* (*right*)

Given these potential effects, interest in and research on activity trackers has grown considerably, resulting in theoretically informed design strategies to increase exercise (e.g., Fogg 2003; Consolvo et al. 2009; Gouveia et al. 2016). Two of the most prevalent techniques used by activity trackers are *self*-*monitoring* and *reinforcing*. For instance, *Fitbit* supports self-monitoring through a dashboard that provides summaries of the data collected (e.g., steps taken a day, a month) (see Fig. 1). The notion is that monitoring provides people with insights into their everyday behavior, such as low activity levels, which in turn motivates them to change. In addition, monitoring is crucial to setting specific goals. Instead of vaguely trying “to walk more”, people can now “walk 3000 steps per day” and monitor their success throughout the day. This should increase the probability of achieving goals (e.g., Gollwitzer 1999). Many trackers further award “badges”, when a goal is achieved—they explicitly *reinforce*. To increase the reward-value of badges, they are often shared among peers or earned through competition.

While extensively used by activity trackers, self-monitoring and reinforcing are among the most primitive motivational mechanisms psychology has in stock. In daily life, these mechanisms may provide some initial effects. However, those effects may not necessarily sustain. Even worse, trackers may even have adverse effects in the long run. For instance, an experimental study (Etkin 2016) found, that though counting steps led participants to walk more, it decreased people’s enjoyment of walking. Etkin (2016) argued that by invoking the metaphor of “measuring” and highlighting quantitative outcomes, attention is drawn away from the intrinsic joys of an activity towards external rewards (Deci et al. 1999). Exercise is experienced a little more like work, which in turn may decrease the likelihood of continued engagement in one’s free time. In other words, instead of supporting people with construing exercise as an enjoyable and meaningful activity to guarantee prolonged engagement, activity trackers establish mechanisms, which guarantee a short-term increase in activity at the risk of potentially detrimental long-term effects.

Surprisingly, studies of how activity trackers are incorporated into everyday life are rare. In this paper, we aim at complementing the mostly behavior-focused, effects-oriented research (Fritz et al. 2014; Rooksby et al. 2014; Epstein et al. 2016) with a study of the *experiential side* of activity tracking as a daily practice. Experience is a rich, dynamic phenomenon, weaving together perception, emotion, motivation and action. A number of conceptual models have been proposed to describe experience in a way to make it accessible for the design of technology. Some models focus on the unique attributes of experience as a phenomenon. For instance, McCarthy and Wright (2004) decomposed the process of experiencing into six sub-processes, from anticipation to reflection and recounting. Forlizzi and Battarbee (2004) described how experiencing, as an ever-present stream of “self-talk”, is transformed into “an experience”, something memorable, a story, a narrative that can also be communicated to others. Other models attempt to characterize the content, or the qualities, of particular experiences. One approach is to focus on the psychological needs fulfilled through a particular experience (e.g., Hassenzahl et al. 2013). Hassenzahl et al. (2010) adapted Sheldon et al.’ s (2001) list of psychological needs and showed that the fulfillment of these needs is linked to positive experiences with interactive products, as well as, to their perception and evaluation. Later work has further expanded on these insights and understandings (e.g., Partala and Kallinen 2012; Tuch et al. 2013; Hassenzahl et al. 2015; Partala and Kujala 2015).

Since we tie our model of experience to need fulfillment, the present study is also a study of how subjective wellbeing is made through technology (see also Calvo and Peters 2014; Desmet and Hassenzahl 2012). Most definitions of wellbeing assume an affective component (e.g., Diener 2000). People need to have positive—enjoyable and meaningful—experiences throughout the day to be happy and to feel satisfied with their lives (e.g., Howell et al. 2011). Since daily activities are substantially determined by the objects they incorporate (Shove et al. 2012; Verbeek 2005), objects, such as activity trackers, have the power to make activities, such as running, more or less need fulfilling (Howell and Hill 2009; Guevarra and Howell 2015; Diefenbach et al. 2016). An enquiry into the daily experience of exercising with activity trackers is thus also an enquiry into daily wellbeing.

Over a longer period of use, many meaningful and less meaningful experiences will occur. Since we were interested in gaining rich insights into the detailed content of experiences, our approach was to gather descriptions of a recent memorable experiences and to submit them to further qualitative analysis. We assume that focusing on a recent experience provides a window to a defining moment in how an activity tracker shapes daily practices of exercising.

All in all, we collected 133 experiences of participants, who have, of their own volition, purchased one of three commercially available trackers—*Fitbit, Jawbone Up* and *Nike* + *Fuelband* (see Fig. 1). The theoretical lens of need fulfillment allows to categorize and understand experiences by tracing their emotional value (positive or negative) back to the fulfillment or deprivation of needs, such as relatedness, autonomy, competence and others. Furthermore, we complement our quantitative findings with rich qualitative insights to better capture the complexity of the experiential side of activity tracker adoption (or abandonment) over time.

## Methods

### Participants and Procedure

Participants were recruited for our online survey through Amazon Mechanical Turk. They received 1.50 USD as compensation. In the survey, we asked participants about their experiences with one out of three wearable activity trackers—*Fitbit*, *Jawbone UP* and *Nike* + *Fuelband*—as well as their practices during initial and prolonged use. Over the course of three weeks, 348 individuals participated. 177 of these completed the survey (retention rate: 51 %). Of those, 44 responses were either vague or duplicate, leaving a total of 133 complete responses for further analysis.

All participants lived in the US (median 30y, 35 % female). Forty-nine participants (37 %) owned a *Fitbit*, while 29 (22 %) owned a *Jawbone Up* and 55 (41 %) a *Nike* + *Fuelband*. The average time of ownership was 8 months. Thirteen participants (10 %) owned the tracker for a month, 33 (25 %) for two to 3 months, 28 (21 %) for 4–6 months, 43 (32 %) for 7–12 months, 15 (11 %) for 1–2 years. One participant owned her tracker for three years.

### Questionnaire

The online questionnaire consisted of two parts: (1) a report of a memorable experience followed by (2) ratings of need fulfillment and usage over time (see Table 1 for an overview). In line with previous studies (Hassenzahl et al. 2010; Sheldon et al. 2001; Tuch et al. 2013; Partala and Kallinen 2012; Karapanos et al. 2009), we asked participants to bring to mind and write down a recent outstanding experience they had with their activity tracker and to provide context to it (e.g., how long ago did the experience occur? Where have you been when the experience occurred? Who was present?).

**Table 1:** Questionnaire

*Memorable experience*
Experience
1 Open question: “Bring to mind a single outstanding positive experience you have had recently with the tracker”
Need fulfillment
30 Closed questions measuring the intensity of need fulfillment for 10 universal human needs proposed by Sheldon et al. (2001) namely autonomy, competence, relatedness, meaning, physical thriving, pleasure, luxury, security, self-esteem, popularity, response from “very slightly or not at all” (1) to “extremely (5), such as: “During this experience I felt I was very capable in what I did”, “During this experience I felt a strong sense of physical well-being”
Affect
20 Closed questions, response from “very slightly or not at all” (1) to “extremely (5), such as: “During this experience to what extent did you feel proud”, “During this experience to what extent did you feel afraid”
*Usage over time*
Expectation phase
1 Open question: “What were your initial motivations and expectations when purchasing the tracker?”
1 Closed question, response from “very slightly or not at all” (1) to “extremely” (5): “To what extent did you expect the tracker to impact your life style?”
Initial use
1 Open question: “Using simple, easy-to-understand terms, what did you initially use the tracker for?”
1 Closed question, response from “very slightly or not at all” (1) to “extremely” (5): “To what extent do you think the tracker fulfilled your initial expectations?”
2 Closed questions, response from “Never” (1) to “Always” (5): “In the first weeks of use, how often did you wear the tracker?”; “In the first weeks of use, how often did you look at/reflect upon the collected information?”
Prolonged use
1 Open question: “Using simple, easy-to-understand terms, what do you currently use the tracker for?”
2 Closed questions, response from “Never” (1) to “Always” (5): “Currently, how often do you wear the tracker?”; “Currently, how often do you look at/reflect upon the collected information?”

Participants were then asked to rate the intensity of need fulfillment in this experience with Sheldon’s et al. (2001) needs questionnaire (see Table 2 for definitions), which captures ten human needs, such as autonomy (i.e., feeling that one is the cause of his or her own actions rather than feeling that external forces or pressure are the cause of his or her action) or competence (i.e., feeling that one is very capable and effective in his or her actions rather than feeling incompetent or ineffective). While Hassenzahl et al. (2010) and Tuch et al. (2013) excluded self-esteem, physical thriving and luxury, we decided to include them as we found them potentially relevant to trackers. Each need was measured with three items. Internal reliability of all scales was satisfactory (see Table 3, diagonal). Accordingly, we computed scale values for each need by averaging the respective items. Table 2 shows the interscale correlations. While in many cases substantial (average interscale correlation = 0.41), the interscale correlations were always smaller than the internal consistency. In other words, while needs may not be completely independent of each other, it is still tenable to understand them as conceptually different.

**Table 2:** Psychological needs, their description (Sheldon et al. 2001), and the questionnaire items

Need	Description	Questionnaire items “*During this experience I felt…*”
Autonomy	Feeling like you are the cause of your own actions rather than feeling that external forces or pressure are the cause of your action	That my choices were based on my true interests and values Free to do things my own way That my choices expressed my “true self”
Competence	Feeling that you are very capable and effective in your actions rather than feeling incompetent or ineffective	That I was successfully completing difficult tasks and projects That I was taking on and mastering hard challenges Very capable in what I did
Relatedness	Feeling that you have regular intimate contact with people who care about you rather than feeling lonely and uncared of	A sense of contact with people who care for me, and whom I care for Close and connected with other people who are important to me A strong sense of intimacy with the people I spent time with
Self-actualization	Feeling that you are developing your best potentials and making life meaningful rather than feeling stagnant and that life does not have much meaning	That I was “becoming who I really am” A sense of deeper purpose in life A deeper understanding of myself and my place in the universe
Physical thriving	Feeling that your body is healthy and well-taken care of rather than feeling out of shape and unhealthy	That I got enough exercise and was in excellent physical condition That my body was getting just what it needed A strong sense of physical well-being
Pleasure-stimulation	Feeling that you get plenty of enjoyment and pleasure rather than feeling bored and understimulated by life	That I was experiencing new sensations and activities Intense physical pleasure and enjoyment That I had found new sources and types of stimulation for myself
Luxury	Feeling that you have plenty of money to buy most of what you want rather than feeling like a poor person who has no nice possessions	Able to buy most of the things I want That I had nice things and possessions That I got plenty of money
Security	Feeling safe and in control of your life rather than feeling uncertain and threatened by your circumstances	That my life was structured and predictable Glad that I have a comfortable set of routines and habits Safe from threats and uncertainties
Popularity	Feeling that you are liked, respected, and have influence over others rather than feeling like a person whose advice or opinion nobody is interested in	That I was a person whose advice others seek out and follow That I strongly influenced others’ beliefs and behavior That I had strong impact on what other people did
Self-esteem	Feeling that you are a worthy person who is as good as anyone else rather than feeling like a ‘‘loser”	That I had many positive qualities Quite satisfied with who I am A strong sense of self-respect

**Table 3:** Interscale correlations of the 10 needs

	Aut.	Com.	Rel.	Mea.	Phy.	Plea.	Lux.	Sec.	Self.	Pop.
Autonomy	(0.76)									
Competence	0.47**	(0.71)								
Relatedness	0.37**	0.27**	(0.91)							
Meaning	0.51**	0.48**	0.53**	(0.88)						
Physical thriving	0.38**	0.45**	0.21*	0.34**	(0.81)					
Pleasure	0.44**	0.49**	0.40**	0.62**	0.52**	(0.68)				
Luxury	0.34**	0.15	0.40**	0.49**	0.10	0.21*	(0.71)			
Security	0.52**	0.29**	0.40**	0.65**	0.15	0.35**	0.66**	(0.80)		
Self-esteem	0.57**	0.36**	0.16	0.40**	0.43**	0.39**	0.44**	0.49**	(0.78)	
Popularity	0.46**	0.29**	0.69**	0.52**	0.27**	0.38**	0.50**	0.53**	0.44**	(0.80)

* p < 0.05, ** p < 0.01

Experienced affect was measured with the PANAS (Watson et al. 1988). It uses 20 verbal descriptors, namely active, alert, attentive, determined, enthusiastic, excited, inspired, proud, strong and interested for positive affect (PA), and afraid, scared, nervous, jittery, irritable, hostile, guilty, ashamed, upset, and distressed for negative affect (NA). Internal reliability was excellent for positive affect (Cronbach’s Alpha = 0.91) and very good for negative affect (Cronbach’s Alpha = 0.87). Accordingly, we computed scale values for positive and negative affect by averaging the respective items. The interscale correlation was −0.21, p < 0.05. While significant, the interscale correlation was low and below the internal consistency, which justifies treating positive and negative affect as distinct concepts.

Measurement of needs and affect was followed by a number of questions, which specifically inquired into how the use of the trackers varied between initial use and prolonged use (see Table 1, section “Usage over time”). With recent findings suggesting that over a third of owners discard activity trackers in the first 6 months of use (Ledger and McCaffrey 2014), we wanted to inquire into this further. To do so, we differentiate the frequency with which users wear the tool (i.e., the wrist-band) from the frequency with which users look at and reflect upon the collected information (i.e., either through web tools or mobile applications).

### Data Analysis

Each of the 133 experiences (narratives) had an average length of 82 words (median = 75, SD = 39). We submitted them to a qualitative content analysis (Adams et al. 2008) to study how activity trackers address one or more of the ten psychological needs. Subordinate concepts within each need category were later identified using open coding (Adams et al. 2008). In addition, we examined participants’ ratings for each reported experience with respect to the saliency of each need (Sheldon et al. 2001).

From the qualitative accounts of participants’ expectations (see Table 1), we noticed that participants’ motivations for purchase and use varied considerably, which in turn may affect their usage and experience. To account for this, we analyzed the qualitative data regarding users’ motivations for purchasing the tracker. This analysis was able to identify two broad groups of users. The *purposive* group (N = 104) purchased the tracker deliberately to achieve a healthier lifestyle (55 %), to quantify and track their physical activity (27 %), or to overcome barriers to exercise (18 %) (e.g., lack of motivation, time and fun associated with exercise). The *explorative* group (N = 29) either received these trackers as a gift (62 %), purchased them impulsively due to their trust in the brand or interest in the tracker’s design (24 %), or purchased them to support friends and family in their goal of achieving healthier lifestyles (14 %).

## Findings

We start with a comparison of the initial versus the prolonged use for the *purposive* and the *explorative* group, focusing on the *frequency of checking* the (online) feedback and the *frequency of wearing* the tracker. We then analyze reported experiences in detail to better understand the needs trackers fulfill and to establish differences across the two user groups as well as over time.

### How Did Usage Change Over Time?

#### Frequency of Checking the Feedback

**Fig. 2:**
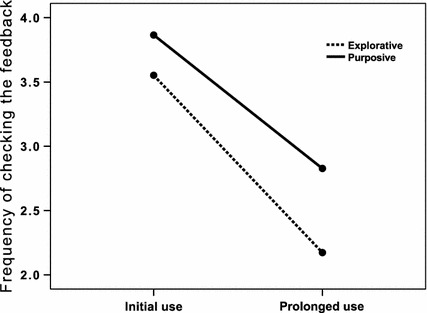
The frequency of checking the online feedback decreased over time for both usage groups, the explorative and purposive usage

A repeated measures ANOVA with estimated *frequency of checking the feedback* as the dependent variable, *time* (initial versus prolonged use) as a within-subject variable, and *use* (explorative versus purposive) as a between-subjects variable revealed a significant main effect for *time*, *F* (1131) = 64.0, *p* < 0.001, *h*_*p*_^*2*^ = 0.33. The estimated frequency of checking decreased from an average of 3.8 on a 5-point scale to an average of 2.7 on a group level. In addition, a main effect of *use* emerged, *F* (1131) = 8.0, *p* < 0.01, *h*_*p*_^*2*^ = 0.06. Purposive users checked the feedback more often (M = 3.4) than explorative users (M = 2.9). The interaction between time and use remained insignificant (see Fig. 2).

The participants provided a number of reasons able to explain the decrease in checking the feedback over time. First, participants found the information provided to increasingly *lack meaning* (N = 8). As one participant noted: “*There’s definitely the idea that collecting data is useful, but after a while, you figure out that numbers are just numbers. I did notice that when I drink more coffee I tend to go to sleep later and not sleep very well. Fitbit didn’t tell me any of that, it’s just a locker for all my habits and activities”* [P91].

Second, participants reported to miss *support for user*-*initiated logging* (N = 4), such as logging one’s food intake, activities and body measurements, blood pressure and glucose. This led to a decrease in the use of the online tool over time: “*I found myself frequently forgetting to input a meal or note the time I ran 12 blocks because I was late for a meeting. At that point, it started to feel like all my data was bunk and incomplete, so I just ditched it all and went back to tracking steps*” [P93]. This emphasizes the need for data completeness. When a complete account of own behaviors was not attainable, the perceived value of the relevant of the tracker appeared diminished.

Third, participants reported an increased *sense of accomplishment* (N = 4), leading to a decreased reliance on the tool to achieve their goals: “*I don’t look at it that much anymore. It got me going in the first months then it was all up to me. I keep walking a lot, I just needed an initial push”* [P89]. This highlights the “scaffolding” nature of current trackers (Gouveia et al. 2015): while they may support overcoming initial motivational problems, they become obsolete, the moment an appropriate practice is established.

While the former two reasons hint at trackers’ potential shortcomings in supporting behavior change, the latter reason suggests that a reduced frequency of checking the feedback is an expected outcome and even implies successful adoption of healthier practices. Next, we explore whether this decrease in the frequency of checking the feedback goes hand in hand with a decrease in the frequency of wearing the tool.

#### Frequency of Wearing the Tracker

**Fig. 3:**
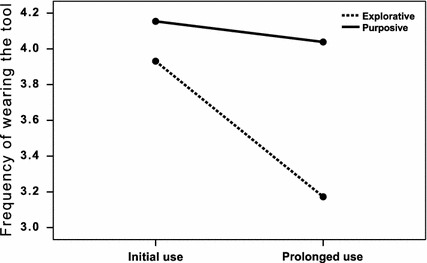
The frequency of wearing the tool decreased over time for the explorative but not for the purposive usage group

A repeated measures ANOVA with estimated *frequency of wearing the tool* as the dependent variable, *time* (initial versus prolonged use) as a within-subject variable, and *use* (explorative versus purposive) as a between-subjects variable revealed a significant main effect for *time,F* (1131) = 11.1, *p* < 0.001, *h*_*p*_^*2*^ = 0.08. The frequency of wearing the tool decreased from an average of 4.1 on a 5-point scale to an average of 3.9 in prolonged use. In addition, a main effect of *use* emerged*, F* (1131) = 11.5, *p* < 0.001, *h*_*p*_^*2*^ = 0.08. This effect was further qualified by a significant interaction of *time* and *use*, *F* (1131) = 4.7, *p* < 0.05, *h*_*p*_^*2*^ = 0.05. Pair-wise t tests revealed significant differences in frequency of wearing the tracker for the explorative (*initial use: M* = 3.9*, SD* = 0.8*, prolonged use: M* = 3.2*, SD* = 1.3*, t* (28) = 2.9, *p* < 0.01) but not for the purposive usage group (*M* = 4.2, *SD* = 1.0, *prolonged use: M* = 4.0*, SD* = 0.9*, t* (103) = 1.0*, p* > 0.10) (see Fig. 3).

Our qualitative analysis revealed a number of reasons why purposive users (who constituted the majority of our sample) reduced the frequency of checking data, but still keep on wearing the tracker.

First, we found that people kept wearing the tracker due to the potential *future value of data accumulation*. As one participant noted: “*I used to look at my data much more than I do now. Now I just keep track of things. I guess I don’t want to lose all this information since it really helped me move my ass around. You never know when it’ll become handy, maybe I’ll show it to my doctor at my next checkup!”* [P131].

Second, while the online feedback lost its significance over time, the primary value of the tracker was in maintaining awareness and re-assuring users about their current level of physical activity: “*(…) I keep it on to check how I’m doing right now. I simply need to press a button to get an overall idea of how I am. It’s much simpler than having to look at a bunch of information and thinking about it*”.

Third, the mere act of wearing the tracker empowered users as it enhanced their sense of self: “*I keep tracking my data which is a confidence booster because it backs up the belief in my mind that I’ve done enough exercise*” [P95].

### Which Needs are Fulfilled by Wearable Activity Trackers?

Table 4 (columns 2 and 3) shows the mean saliency (95 % Confidence Interval) of the experienced need fulfillment for each of the ten psychological needs. *Physical thriving*, *autonomy* and *competence* were the most salient needs with mean ratings higher than four on a five-point intensity scale. They were followed by *pleasure*-*stimulation, self*-*esteem and self*-*actualization*–*meaning. Popularity* and *relatedness* constituted the third group, while *security* and *luxury* were the group of the least salient needs with mean ratings of three and lower on the five-point scale.

**Table 4:** Mean ratings for each need, 95 % confidence intervals, partial correlation with positive affect (PA) while controlling for negative affect and with negative affect (NA) while controlling for PA, and loadings on the two components resulting from PCA

Need	Mean	CI (95 %)	Corr_PA_	Corr_NA_	C1	C2
Physical thriving	4.17	4.03–4.28	0.31**	−0.01	0.78	
Autonomy	4.03	3.88–4.13	0.20*	−0.14	0.66	
Competence	4.08	3.92–4.13	0.31**	−0.16	0.72	
Stimulation	3.83	3.69–3.95	0.35**	−0.6	0.68	
Self-esteem	3.79	3.61–3.92	0.30**	−0.01	0.71	
Meaning	3.43	3.22–3.61	0.28**	0.12	0.51	0.63
Popularity	3.15	2.94–3.32	0.26**	0.07		0.84
Relatedness	3.13	2.92–3.33	0.20*	0.04		0.91
Security	3.00	2.81–3.16	0.10	0.07		
Luxury	2.53	2.43–2.79	0.08	0.12		

* p < 0.05, ** p < 0.01, component loadings <0.30 were suppressed

All needs but security and luxury had a significant positive correlation with positive affect while controlling for negative affect (Table 4, column 4). Since later qualitative findings also revealed minimal relevance of security and luxury in users’ experiences with the trackers, these two categories were excluded from further analyses.

The simple bivariate correlations among needs (see Table 2) already hint at potential groups of relevant needs when experiencing activity trackers. A second-order Principal Components Analysis (PCA) of the eight needs with Varimax (orthogonal) rotation revealed a two-dimensional structure. Component 1 consisted of *physical thriving, competence, self*-*esteem, stimulation* and *autonomy* and accounted for 50 % of the variance in participants’ ratings. Component 2 consisted of *relatedness*, *popularity* and *meaning* and accounted for 14 % of the variance in participants’ ratings (Table 4, column 5 and 6, for the loadings of the eight needs on the two components; Fig. 1 for a plot).

**Fig. 4:**
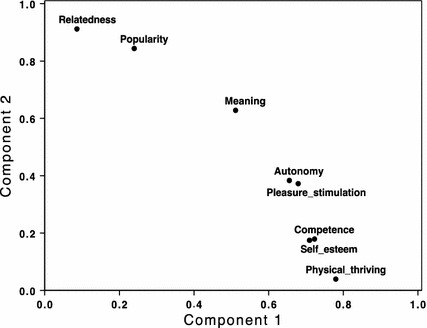
Relationships among needs

All in all, one can clearly distinguish two sources of meaningful experience. One is not surprisingly fueled by the experience of physical thriving. Success is then mainly marked by feelings of competence and self-esteem. However, positive experiences can also be motivated socially by relatedness and popularity. Meaning is in between both, underlining its rather universal nature (Fig. 4).

In the remainder of the section, we qualitatively analyze the reported experiences. Our goal is to elaborate the ways in which trackers address human needs and to further inquire into the differences between explorative and purposive use as well as between initial and prolonged use. While we knew for how long each participant owned the tool, the majority of participants did not provide any information about when the reported experience took place. However, in most cases this could be inferred from their qualitative accounts. We classified each provided narrative as being either (a) an experience which took place during initial use or (b) an experience which took place during prolonged use. This was done on the basis of explicit references to time (e.g., “I was initially shocked as I realized how inactive I was…”). Two researchers performed this categorization independently. Interrater agreement was satisfactory (Cohen’s K = 0.71). Cases of disagreement were either resolved jointly by the two researchers or removed from the dataset. Overall, 57 (43 %) of experience narratives related to initial use and 62 (47 %) to prolonged use. For 14 narratives (10 %) it was impossible to infer a category.

### Experiences of Physical Thriving, Competence and Self-Esteem

Initial experiences among the exploratory group, were often marked by dismay: *The biggest shock was actually seeing how much I sit. I was completely unaware that I spend 8*-*9* *h per day at a desk! I completely overestimated how much exercise I got into my days*” [P58], or “*I’ve never worried about my health until getting my Fuelband. I’ve always thought I was in perfect shape but boy was I wrong. (…) It was a wake*-*up call. You only realize after putting a number on these things*” [P44]. Suddenly, people realized how little they actually walk. This impacted how they felt about their bodies. As one participant noted: “*Even though I have a slim silhouette, I felt, to put it bluntly, fat. I never thought I was so unhealthy (…) it made me think about my family. If I’m unfit, they’re even in worse shape!*” [P56].

In contrast, participants from the purposive group already had a more or less realistic sense of their current activity levels, *e.g.,* “*Seeing my initial numbers wasn’t surprising, I kind of expected it (…) well, I did buy it to become healthier, so I kind of knew I would be starting from the bottom*” [P7].

Over time, as participants started to achieve or even surpassed their daily activity goals, they reported that the feedback influenced the perception of their health and wellbeing: “*Seeing the numbers stack up makes me feel healthier*” [P20], “*(…) having knowledge that I am getting enough exercise every day makes me feel good about myself. It also keeps my body feeling good and if you feel good then your day is good*” [P57].

Overall, the narratives suggest that feelings of physical thriving, competence and self-esteem tend not to wear off even after extended use and were strongly related to goal-setting: “*(…) Hitting my goals day after day makes me feel more able to achieve a healthier lifestyle*” [P50].

### Autonomy and Meaning

We found activity trackers to profoundly shape participants’ experiences through empowering them to make necessary changes in their lives that were previously thought of as impossible or difficult. This provided a sense of autonomy, a feeling to be the cause of one’s actions. As one of the participants commented:

“*I was sitting in a chair on the pool deck watching my daughter swim this morning because I didn’t feel like swimming today. I was feeling too guilty about just sitting and realized there was no reason I couldn’t just walk laps around the pool while she swam. I found that it took 15 laps to equal 1000 steps! I realized I can do this every morning (or afternoon or whenever) for the rest of the summer when she wants to swim. I can do it in my bathing suit and then just jump in the pool afterward and swim with her for two more exercises. I had 5000 steps before noon today* - *really good for a Saturday!”* [P4].

In some instances, participants remarked how the tracker supported them in developing their best potential and making life meaningful through enabling them to attain their ideal self, *e.g.,* “*After about 6* *months I started to notice a completely different person when I looked in the mirror and I owe it all to the Fuelband*” [P12], “*(…) my BMI level is perfect (…) it’s a rewarding feeling to accomplish something that I used to think was impossible*” [P3]. In addition, it inspired a deeper understanding of own behavior, *e.g.,* “*(…) it helped me reach a state where I really understand myself*” [P93].

### Popularity and Relatedness

As we mentioned earlier, social aspects constituted a distinct second component in users’ experiences with the activity trackers. Trackers supported the users’ need for relatedness in a number of ways. First, participating in the online community provided a sense of belonging and social support, as individuals would interact with others that had similar goals and faced similar challenges. As one participant noted:

“*it’s encouraging to be connected to people all over the world and to see their daily activity and sleep patterns. To know that I am not the only one having a bad day now or then or that my sleep patterns are not that crazy*” [P33].

Second, trackers became the pivotal element of direct social support. Some participants reported purchasing a tracker to support a family member or close friend in overcoming his or her weight problem. Using the tracker and going through this effort together provided them with a sense of *closeness*, as one participant said:

“*[I got the tracker] to support my younger sister, who was struggling with trying to lose weight (…) After a day or so we stopped comparing daily goals because I did not want to discourage her. We still wore our bands, and after 2* *months we did a major weigh in (…) sharing this experience with my sister has been a lot of fun*” [P112].

Last, we found that the trackers stimulated feelings of popularity and social affirmation, when others would show interest in it, *e.g.,* “*I have recommended it to all my friends! It’s nice when they actually listen to my advice instead of criticizing me*” [P132], as well as individuals, who would compete and try to outperform others, *e.g.,* “*I love moving up in the rankings, they’ll have to try harder to beat ME! (…) they keep asking me how I manage (to overtake them)”* [P35].

### Stimulation

Finally, similar to prior work that has highlighted playfulness (Lucero et al. 2014) and stimulation (Diefenbach et al. 2014) as crucial experiential qualities in the adoption of interactive products, participants often remarked on the ludic character of the trackers and the pleasure derived either from the act of tracking one’s behaviors, *e.g.,* “*I love being able to see how far I’ve come from when I wake up in the morning to when I go to sleep at night*” [P12], or from turning an activity experienced as chore into fun through game mechanics, *e.g.,* “*I enjoyed getting a ‘score’ for each one of my workouts (…) I thought it looked really fun, which is not something I usually associate with getting fit*” [P115].

## Discussion

Our study revealed a broad two-dimensional structure of users’ experiences with activity trackers— either driven by physical thriving or relatedness—and a more nuanced picture of the adoption of trackers in daily life. Most importantly, while reflection through feedback lost its relevance over time, at least purposive users continued to wear the tracker and to derive value from it.

The study showed that tracking has a nuanced social component (i.e., relatedness, popularity, see also Rooksby et al. 2014, and Maitland et al. 2006). In fact, socially motivated experiences ranged from belonging and social support provided through the online communities, to the stronger more direct social exchange among family members, when they purchased a tracker for a relative and joined in their efforts towards a better, healthier self. This more cooperative and supportive mode is very rarely taken up by commercially available trackers. They rather employ competition (i.e., fulfilling popularity rather than relatedness) as the primary mode of social exchange, supposedly borrowed from the professional world of sports.

Obviously, a crucial question for the design and relevance of activity trackers is whether and how they support behavior change in prolonged use. While a recent study found that over a third of owners of wearable activity trackers discarded them within 6 months (Ledger and McCaffrey 2014), others (e.g., Fritz et al. 2014) found that although many people eventually stop using them, some keep on deriving value and motivation even after many months or years of use. In line with the latter, our study revealed that while reflection inspired by numerical feedback lost its relevance over time, users (at least from the purposive group) continued to wear the tool, implying a gradual and successful appropriation rather than creeping rejection. Sustained use of the tracker depended on different aspects, ranging from the *perceived future value of data accumulation*, to a gradual shift towards *opportunistic engagement* with the reductive wrist-worn display, as well as the mere symbolic *empowerment* that users felt when wearing the tool (see Karapanos 2015, for a list of design strategies to sustaining engagement).

While the experience seems rich, an open question is certainly whether all this went hand in hand with the formation of healthy habits. While we are in no position to answer that directly, we believe the extended engagement to be an important prerequisite of the formation of habits. Our findings suggest that whereas individuals initially relied on the tracker to create awareness of their physical activity, over time, they acquired an “understanding” of their own daily activity levels and how to achieve them. As one participant pointed out: *“I learned that it’s not that hard to walk more… by changing little things in my life, the steps kept summing up and currently I just wear it. I don’t need to look at it and reflect on the information as I did in the beginning*” [P41]. Moreover, the fact that needs for self-esteem and competence were salient, hints at a successful adoption of trackers, which should be accompanied by increased levels of activity. It seems far-fetched to assume that feelings of achievement and competence do not correspond to changes in behavior, given the numerical feedback provided by the trackers. All in all, these results suggest that a decrease in users’ daily involvement with feedback data does not necessarily imply a failure in supporting behavior change.

The differences between purposive and explorative users emphasize the importance of self-set goals prior to usage. While for purposive users the motivational goal (e.g., to lose weight, to be fitter) is already existent, explorative users are often not even correct in the perception of their own levels of physical activity. While the tracker just confronts explorative users with this “truth”—unblinkingly, in the disguise of a seemingly neutral number—initial experiences turn negative, marked by dismay. While some took this as the wake-up call needed to inspire change, for others this led to reduced self-esteem, feelings of incompetence and to the gradual disengagement with the tracker. This points at a common problem with activity trackers and self-quantification in general. Their rather neutral stance does not support users with the implementation of goals (Hassenzahl and Laschke 2015). In other words, while the tracker will reveal that a person doesn’t walk enough, the very same person cannot expect much support in findings ways of how to incorporate walking into daily routines (see Laschke et al. 2014, for an alternative strategy). For purposive users this is not be a problem, since those users actively search for related information, but for an explorative user this can constitute a serious barrier to adoption and ultimately to change. The story about walking laps around the pool mentioned above is an illustrative example. This user discovered a personally meaningful way of smuggling a couple of extra steps into a situation not formerly connected to activity. Others may not be as inventive. Trackers should support users better in identifying opportunities for activity in everyday life.

In general, commercially available activity trackers tend to ignore knowledge from the domain of motivational psychology (Conroy et al. 2014). Exceptions are simple challenges and badges as rewards. *Nike*+ , for example, puts out a triple challenge to lure its users into running three times a week. However, this may even have a detrimental rather than facilitating effect (Fritz et al. 2014). Research found that extrinsic motivations, such as external rewards (i.e., a badge, a cookie), can replace intrinsic motivation for an activity, thereby leading to a decrease in their engagement with the activity once the extrinsic rewards are removed (e.g., when a badge loses its appeal) (Deci et al. 1999; see also Etkin 2016).

On the other hand, our study suggests that for some, numerical feedback can be rewarding (e.g., “*seeing the numbers stack up makes me feel healthier*”). This impact was apparent even after prolonged use and, in some cases, participants pointed out a link between the use of the tracker and observable changes in their physical health (e.g., *“I always knew I could lose the extra pounds* (*…*) *It feels good to prove everyone wrong and stop feeling like a loser”* [P17]). We believe that rewards can be more effective in the long run, if they are more tightly coupled to actual meaningful and achievable goals and observable changes in users’ physical health. To get a badge for “running three times a week”, “6 months on a row” on “Halloween” or for “10 k Nike fuel” seems too arbitrary to be effective. And if this appeals, it might even undermine the actual overarching goal, by making exercise contingent on arbitrary extrinsic rewards rather than intrinsic goals of being more healthy and happy.

It is fair to conclude that the intricacies of the experiential side of prolonged activity tracker use let the usage scenarios envisioned by commercial vendors appear overly simplistic and naïve. As with medicine, a proven-effects approach seems predominant. However, activity trackers as they are and as they could be more than just “tools” to change behavior. Through incorporation in daily life, they offer new social experiences, new ways of boosting self-esteem and getting closer to our ideal selves. It is not only about health and loss of weight. The experiential perspective reveals how activity trackers become pivotal for establishing practices of health, which will increase individual wellbeing. It is important to understand that improving one’s activity levels is not an end in itself. It is simply the means towards increasing one’s self-esteem, health and body perception, competence, and ultimately happiness in life. Collecting and understanding the experiences that activity trackers mediate, how these unfold over time, is critical for a better understanding of how to effectively stimulate behavior change, as well as for a richer understanding of the psychological benefits, these tools have and could have in real life.
